# Validity and reliability of a trigonometry-based method for the measurement of tooth movement on digital models

**DOI:** 10.1590/2177-6709.26.3.e2119148.oar

**Published:** 2021-06-30

**Authors:** Renata de Faria SANTOS, Bruno Fernandes de Oliveira SANTOS, Victor Miranda FERNANDES, Luciana Duarte CALDAS, Taiana de Oliveira BALDO, Gladys Cristina DOMINGUEZ

**Affiliations:** 1 Universidade de São Paulo, Departamento de Ortodontia (São Paulo/PR, Brazil).; 2 Universidade Federal de Sergipe, Hospital Universitário (Aracaju/SE, Brazil).; 3 Universidade Federal da Paraíba, Departamento de Informática (João Pessoa/PB, Brazil).; 4 Universidade Federal do Rio de Janeiro, Departamento de Ortodontia (Rio de Janeiro/RJ, Brazil).

**Keywords:** Dental models, Tooth movement techniques, Odontometers, Rotation, User-computer interface

## Abstract

**Objective:**

The objectives of the present study were to develop a method for longitudinally measuring tooth rotation, inclination and angulation on digital models, and to test the method validity and reliability.

**Methods:**

The initial and final planned models of 14 patients treated with Invisalign^®^ (386 teeth) were exported from ClinCheck^®^. The rotation, inclination and angulation values were assessed for the incisors, canines, premolars and molars, in both models, using trigonometry. An application was developed in Python 2.7 to automate the measurements. The ∆planned (variation in the position between the initial and final planned models) was obtained for each tooth and each type of movement. To test the validity, the degree of agreement between the ∆planned and the values available in the Invisalign^®^ Table of Movements was assessed using the Intraclass Correlation Coefficient (ICC) and Bland-Altman analysis. For intra and inter-rater reliabilities, the ∆planned was obtained again.

**Results:**

Excellent ICCs (> 0.9) and limits of agreement with narrow and clinically acceptable discrepancies were obtained for the rotation of all teeth (except maxillary canines, which had broader limits: -3.47 - 5.43) and for the inclination of premolars and molars. The inclination of anterior teeth and angulation of all teeth had ICCs and limits that were not indicative of great agreement. The reliability was high for the three movements (discrepancy <2°).

**Conclusions:**

The method developed is reliable and suitable for longitudinally measuring inclination (posterior teeth) and rotation (except maxillary canines). It has limited value for the other movements measurements.

## RESUMO

**Objetivo:**

Os objetivos do presente estudo foram desenvolver um método para medir longitudinalmente a rotação, inclinação e angulação dentárias em modelos digitais, e testar sua validade e reprodutibilidade.

**Material e Métodos:**

Os modelos inicial e final planejado de 14 pacientes tratados com Invisalign® (386 dentes) foram exportados do ClinCheck®. Os valores de rotação, inclinação e angulação foram calculados para incisivos, caninos, pré-molares e molares, em ambos os modelos, por meio de trigonometria. Um aplicativo desenvolvido em Python 2.7 foi utilizado para automatização das medidas. O ∆planejado (variação na posição entre os modelos inicial e final planejado) foi obtido para os movimentos descritos, nos diferentes grupos de dentes. A validade do método foi verificada pelo grau de concordância entre o ∆planejado e os valores disponíveis na Tabela de Movimentos do Invisalign®, utilizando-se o Coeficiente de Correlação Intraclasse (ICC) e a análise de Bland-Altman. O ∆planejado foi novamente calculado para análise da reprodutibilidade intra e interexaminadores.

**Resultados:**

Excelentes ICCs (> 0,9) e limites de concordância com discrepâncias reduzidas e clinicamente aceitáveis foram obtidos para a rotação de todos os dentes (exceto os caninos superiores, que expressaram limites mais amplos: -3,47 - 5,43) e para a inclinação de pré-molares e molares. A inclinação dos dentes anteriores e a angulação de todos os dentes apresentaram ICCs e limites não representativos de boa concordância. A reprodutibilidade foi alta para os três movimentos (discrepância < 2°).

**Conclusões:**

O método desenvolvido é reprodutível e se mostrou adequado para mensuração longitudinal da inclinação de dentes posteriores e rotação de todos os dentes, exceto caninos superiores. Apresenta valor limitado para as demais mensurações.

## INTRODUCTION

Dental inclinations, angulations, and rotations are essential aspects to be evaluated during orthodontic treatment and are included in Andrews’s six keys to normal occlusion.^1^

Longitudinal evaluations of inclination and angulation between the beginning and conclusion of treatment are typically measured with the aid of lateral head films^2^ ^-^ ^4^ or panoramic radiographs.^5^ ^,^ ^6^ However, only incisor inclination and posterior teeth angulation can be measured using lateral radiographs, while only dental angulation can be assessed in panoramic radiographs. Moreover, superimposition remains a problem in both types of exams.^7^ Although cone-beam computed tomography (CBCT) may overcome these limitations, it is not indicated as a routine exam.^8^

The measurement of dental rotations is a significant factor for predicting posttreatment stability.^9^ Little’s irregularity index^10^ expresses the degree of anterior segment alignment, but has limited value for the expression of rotation because the results are a combination of rotation and inclination.^11^ The American Board of Orthodontics (ABO) introduced the objective grading system (OGS) to evaluate finished cases according to eight criteria.^12^ Among these, the buccolingual inclination is used to indirectly assess the inclination of posterior teeth. Alignment is another criterion, but for the same reasons of Little’s irregularity index, has limited value for expressing rotation reading.

Longitudinal evaluations of dental positioning are important both clinically and scientifically. Some studies have measured tooth inclination and angulation using plaster or digital models in a cross-sectional manner,^7^ ^,^ ^13^ ^-^ ^15^ with the longitudinal assessment being restricted to the use of radiographic exams.

Digital models can allow obtaining angular measurements, both cross-sectionally and longitudinally. Huanca et al.^13^ and Lombardo et al.^16^ described a method based on trigonometry that allowed the assessment of teeth inclination,^13^ angulation,^13^ and rotation^16^ on digital models. The idea of obtaining dental positioning by means of trigonometry seems to be a viable option, but since these methodologies were not validated, the purposes of the present study were (1) to develop a method to longitudinally measure tooth rotation, inclination and angulation on digital models and (2) to test its validity and reliability.

## MATERIAL AND METHODS

This study included the digital models of 14 patients who started orthodontic treatment with Invisalign^®^ (Align Technology, Santa Clara, CA, USA) at the research clinic of the Dental School at *Universidade de São Paulo* (FOUSP). Ethical approval was obtained by Research Ethics Committee (number 2.701.787) of the aforementioned institution, and written consent was obtained from all subjects.

The initial and final planned models (predicted by the software as an estimate of treatment results) were exported from ClinCheck^®^ (Align Technology, Santa Clara, CA, USA). Therefore, for each patient, four models were exported in *Standard Triangle Language* (.stl): two initial (one upper; one lower) and two final planned (one upper; one lower). The initial models were obtained through polyvinyl siloxane impressions.

The methodology development was based on the description of Huanca et al.^13^ for quantifying inclination and angulation, and Lombardo et al.^16^ for rotation. The values were obtained through trigonometry for each tooth in the initial and final models, and angular variation was calculated (∆rotation, ∆inclination, and ∆angulation) for each tooth of the 14 patients (n=386 teeth). An application was developed in Python 2.7 to automate the measurements.

### METHOD FOR THE ACQUISITION OF ROTATION, INCLINATION, AND ANGULATION VALUES

**Figure 1:**
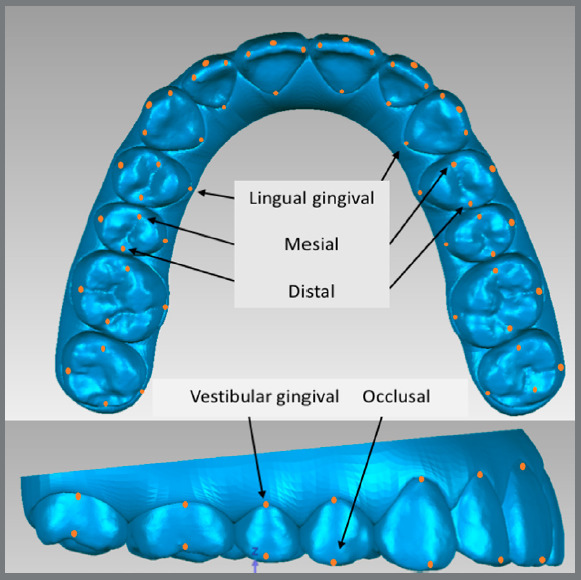
Disposition of points lingual gingival, mesial, distal, occlusal and vestibular gingival.

**Figure 2:**
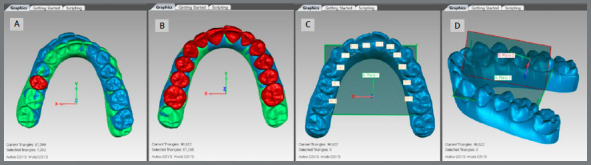
initial model in blue and final planned model in green. A) “Best fit” alignment of unit #15. After the alignment, all the five points of this tooth on the initial model were copied to this same tooth on the final model. B) “Best fit” alignment of all teeth. C) Plane 1 created by the best adjustment between the lingual gingival points of all teeth (except second molars ). D) Plane 2 created perpendicular to Plane 1 and Plane XZ.

The *.stl* models obtained from ClinCheck^®^ were imported into Geomagic Control^®^ (North Carolina, USA) *software.* Five points were marked per tooth on the initial model (Fig 1). Subsequently, the “best fit” alignment was performed for each tooth from the initial model with the respective tooth from the final planned model. The points were thus copied from the initial to the final model (Fig 2A).

#### Establishing the Reference Plane

The establishment of a reference plane is necessary to make angular measurements. A “best fit” alignment of all teeth (except second molars, Fig 2B) was performed. Then, the reference plane was defined on the initial model as the best adjustment of the lingual gingival points of all teeth (Fig 2C) and labeled Plane 1. A median reference plane was created and referred to as Plane 2 (Fig 2D). Planes 1 and 2 were copied to the final planned model.

After this procedure, the Cartesian Space (XYZ) was reoriented so that the XY Plane would coincide with Plane 1 and the YZ Plane, with Plane 2.

#### Rotation measurements

**Figure 3:**
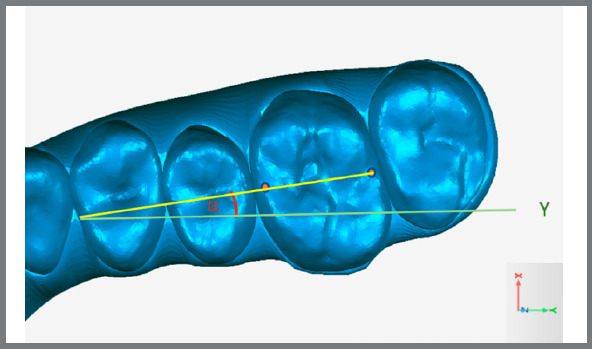
Yellow represents the straight line formed by mesial and distal points, and green, the straight line parallel to Y axis; α represents the angle formed by these two lines.

**Figure 4:**
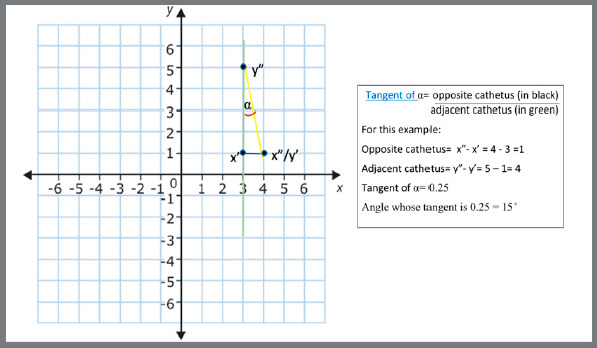
obtaining the rotation measurement by the arctangent function.

Rotation was set as the angle between a line formed by the mesial and distal points of each tooth and the Y-axis (Figs 3 and 4). To minimize the risk of errors and automate the process, angular measurements were not manually performed. The coordinates of each point (X, Y, Z values) were exported from Geomagic^®^ in*.iges* format and imported into the applications.

#### Angulation and inclination measurements

For the measurement of these two angles, to fully capture the movement and not one vector component only, it was necessary to reorient the Cartesian Space for each tooth with the aid of a rotational matrix, resetting the rotation. Thus, the Y axis was rotated to coincide with the line between the mesial and distal points of each tooth, and only after this rotation, the values of angulation and inclination were obtained. This procedure was essential to avoid the influence of tooth rotation on the calculation of inclination and angulation.

**Figure 5:**
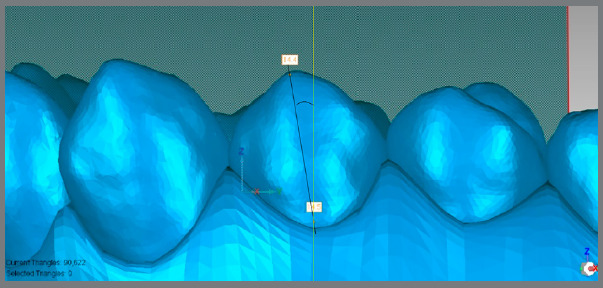
Black represents the line formed by occlusal and vestibular gingival points; yellow represents the Z-axis. The angulation is given in relation to YZ plane.

**Figure 6:**
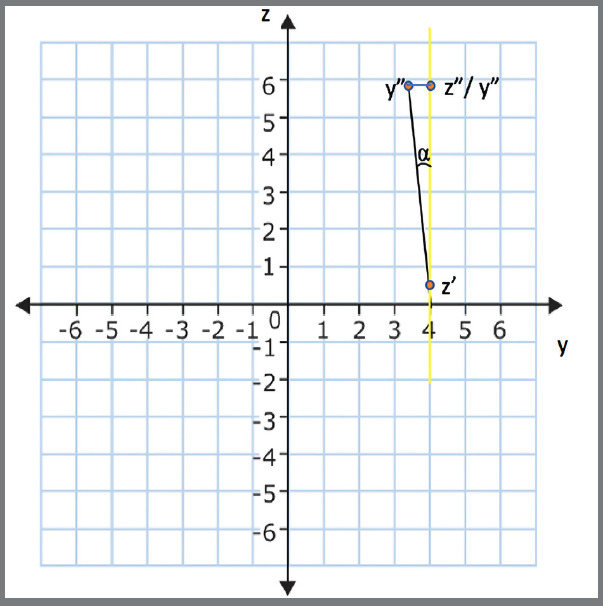
Obtaining the angulation measurement by the arctangent function.

**Figure 7:**
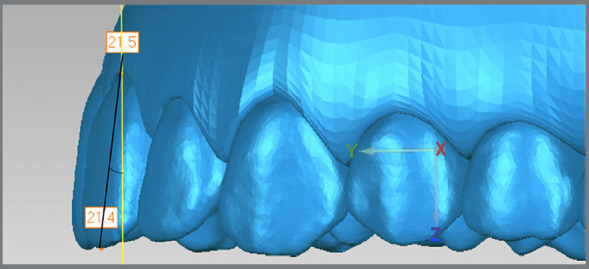
Black represents the line formed by occlusal and vestibular gingival points; yellow represents the Z-axis. The inclination is given in relation to XZ plane. It is also obtained by the arctangent function.

The developed application performed the realignment for each tooth. Given a line between the occlusal point and vestibular gingival point, the value of the angulation corresponded to the angle between the projection of this line on YZ plane and the Z-axis (Figs 5 and 6), while the inclination corresponded to the angle between the projection of the same line on the XZ plane and the Z-axis (Fig 7).

### DEVELOPMENT OF THE APPLICATIONS

Two applications were developed in Python 2.7. The first application extracts the coordinates from *.iges* file exported from Geomagic^®^ and saves the file in *.csv* format, while the second reads the data and performs the measurements. Applications and instructions are available at *http://app.renatadefaria.com.br/IGES%20Coordinates%20extractor/* and *http://app.renatadefaria.com.br/Teeth%20position%20on%20digital%20models/*. In the second application, a *.csv* file (containing the three-dimensional coordinates of each point) was used as an input, and a new *.csv* file was generated, which contained: (1) the degree of rotation of each tooth, (2) the coordinates resulting from the rotational matrix, and (3) the angles of inclination and angulation calculated from the rotated coordinates.

### SAMPLE CHARACTERISTICS

Convenience sample composed of 4 men and 10 women, with a mean age of 31.37 years (range from 20.85 to 57.13) was used. The inclusion criteria were patients undergoing orthodontic treatment with Invisalign^®^ at the research clinic at FOUSP. All of them initially presented a reduced or negative overbite, with a mean value of -0.28 mm (minimum of -2.0; maximum of 1.3), and a mild degree of crowding (< 3.5 mm). An overbite increase was planned for all the patients. 

This study was composed of 386 teeth (194 maxillary; 192 mandibular) divided into the groups: incisors, canines, premolars, and molars (*n* per group in Tables 1 and 2).

**Table 1:** ICC and Dahlberg index for ∆Method x ∆Invisalign Table (maxillary arch).

	Rotation			Inclination			Angulation			
	ICC	CI 95%	D	ICC	CI 95%	D	ICC	CI 95%	D	n
Incisors	0.97*	0.95-0.98	1.13	0.70*	0.53-0.81	3.01	0.72*	0.57-0.83	2.08	56
Canines	0.98*	0.96-0.99	1.72	0.69*	0.43-0.84	2.34	0.53*	0.21-0.75	3.53	28
Premolars	0.99*	0.98-0.99	0.82	0.92*	0.88-0.96	1.47	0.66*	-0.06-0.90	2.88	54
Molars	0.97*	0.96-0.98	0.70	0.91*	0.85-0.95	1.32	0.64*	-0.07-0.88	2.83	56
Total	0.98*	0.97-0.99	1.06	0.82*	0.76-0.86	2.12	0.66*	0.33-0.81	2.75	194

*p<0.05; ICC: Intraclass Correlation Coefficient; CI: Confidence Interval; D: Dahlberg.

**Table 2:** ICC and Dahlberg index for ∆Method x ∆Invisalign table (mandibular arch).

	Rotation			Inclination			Angulation			
	ICC	CI 95%	D	ICC	CI 95%	D	ICC	CI 95%	D	n
Incisors	0.99*	0.99-1.00	0.63	0.71*	0.55-0.82	3.58	0.47*	0.24-0.65	2.71	56
Canines	0.99*	0.98-0.99	1.01	0.82*	0.65-0.91	3.08	0.89*	0.77-0.95	2.19	28
Premolars	0.99*	0.99-1.00	0.76	0.94*	0.90-0.96	2.92	0.86*	0.76-0.92	1.81	54
Molars	0.98*	0.97-0.99	0.45	0.96*	0.94-0.98	0.51	0.60*	0.28-0.77	1.50	54
Total	0.99*	0.99-1.00	0.70	0.83*	0.78-0.87	2.74	0.76*	0.69-0.81	2.09	192

*p<0.05; ICC: Intraclass Correlation Coefficient; CI: Confidence Interval; D: Dahlberg.

For ICC, the sample size calculation was performed considering an α of 0.05, a power of 80% (β = 0.2), and a moderate ICC of 0.7. A sample size of 10 teeth per group was necessary.^17^ For Bland-Altman analysis, the parameters used to estimate the sample size were an α of 0.05, a power of 80% (β = 0.2), an expected mean difference of 3.5°, an expected standard deviation of the differences of 1.7°, and the maximum expected difference of 10°. A sample size of 11 teeth was found to be necessary.

Since ∆rotation, ∆inclination, and ∆angulation variations were obtained between the initial and final planned models of patients undergoing treatment with Invisalign^®^, the proposed method was expected to produce ∆s similar to those presented in the Invisalign^®^’s Table of Movements.

### STATISTICAL ANALYSIS

The validity was verified by the Intraclass Correlation Coefficient (ICC) (2-way random, single measurement, absolute agreement), and by the Bland-Altman analysis of agreement between the ∆ calculated with the method and the ∆ available in the Table of Movements provided by Invisalign^®^. Descriptive statistics of the difference (∆Invisalign Table - ∆Method) were computed, and a 1-sample Student *t*-test was applied to verify the presence of systematic error. The random error was assessed by the Dahlberg formula.

For the intra-rater reliability analysis, 50% of the models were randomly selected and remarked after one month, and the ∆Method was obtained again. The same 50% models were remarked by a second rater for the assessment of inter-rater reliability. The ICC (2-way mixed, single measurement, absolute agreement) and Bland-Altman were used for both analyses. Descriptive statistics of the difference, a 1-sample Student *t*-test, and the Dahlberg formula were also applied.

Variables were expressed as the mean and standard deviation. The normality assumption was investigated by the Kolmogorov-Smirnov test.

## RESULTS

### VALIDITY

Tables 1 and 2 describe the results of the ICC, whereas Tables 3 and 4 describe the P-value for the 1-sample Student *t*-test (comparing the difference between ∆Invisalign and ∆Method to 0), the mean of the difference, the 95% confidence interval for the differences and the Bland-Altman limits of agreement.

**Table 3:** Descriptive statistics, 1-sample t test, and 95% CI values for differences and limits of agreement (positive numbers represent underestimations, and negative represent overestimations of measurements obtained by the method with respect to Invisalign Table). Maxillary arch.

Differences (Invisalign Table - Method)	P value	Mean Difference (degrees)	SD (degrees)	IC 95% (degrees)	Limits of agreement (degrees)	n
Rotation						
Incisors	0.06	0.40	1.57	-0.15 - 0.82	-2.67 - 3.11	56
Canines	0.03*	0.98	2.27	0.10 - 1.86	-3.47 - 5.43	28
Premolars	0.15	0.19	0.91	-0.07 - 0.44	-1.59 - 1.98	54
Molars	0.10	0.16	0.71	-0.03 - 0.36	-1.23 - 1.55	56
Inclination						
Incisors	0.60	0.39	4.29	-0.85 - 1.45	-8.01 - 8.80	56
Canines	0.87	-0.10	3.36	-1.40 - 1.20	-6.69 - 6.47	28
Premolars	0.99	-0.003	1.5	-0.42 - 0.41	-2.94 - 2.94	54
Molars	0.46	0.15	1.47	-0.25 - 0.54	-2.73 - 3.03	56
Angulation						
Incisors	0.36	-0.29	2.34	-0.94 - 0.35	-4.88 - 4.30	56
Canines	0.06	1.73	4.77	-0.11 - 3.58	-9.34 - 11.08	28
Premolars	< 0.001*	3.70	1.60	3.28 - 4.18	0.56 - 6.84	54
Molars	< 0.001*	3.55	1.70	3.10 - 4.00	0.22 - 6.88	56

*p<0.05.

**Table 4:** Descriptive statistics, 1-sample t test, and 95% CI values for differences and limits of agreement (positive numbers represent underestimations, and negative represent overestimations of measurements obtained by the method with respect to Invisalign Table). Mandibular arch.

Differences (Invisalign Table - Method)	P value	Mean Difference (degrees)	SD (degrees)	IC 95% (degrees)	Limits of agreement (degrees)	n
Rotation						
incisors	0.75	-0.04	0.90	-0.28 - 0.20	-1.80 - 1.72	56
canines	0.62	-0.14	1.45	-0.70 - 0.42	-2.98 - 2.70	28
premolars	0.84	0.02	0.61	-0.15 - 0.18	-1.17 - 1.21	54
molars	0.17	-0.09	0.50	-0.23 - 0.04	-1.07 - 0.89	54
Inclination						
incisors	0.22	-0.62	3.63	-1.62 - 0.38	-7.73 - 6.49	56
canines	0.22	0.60	2.53	0.39 - 1.60	-4.35 - 5.55	28
premolars	0.80	0.05	1.45	-0.35 - 0.45	-2.79 - 2.89	54
molars	0.88	0.01	0.72	-0.18 - 0.21	-1.40 - 1.41	54
Angulation						
incisors	0.18	-0.69	3.8	-1.70 - 0.33	-8.14 - 6.76	56
canines	0.65	0.23	2.59	-0.81 - 1.28	-4.84 - 5.30	28
premolars	0.01*	0.79	2.17	0.18 - 1.39	-3.46 - 5.04	54
molars	< 0.001*	1.13	1.82	0.64 - 1.63	-2.44 - 4.70	54

*p<0.05.

Considering the data provided by Invisalign^®^ as the gold standard, the method for calculating rotation was shown to be accurate for maxillary and mandibular teeth because it presented excellent ICC agreement values (greater than 0.9 for all groups) and because the Bland-Altman limits of agreement showed narrow and clinically acceptable (≤ 3º) discrepancies (except maxillary canines). The difference between the method and the Table of Movements was close to 0 (and not significantly different) for each group of teeth, indicating that no systematic bias was found. For maxillary canines, broader limits of agreement (-3.47 - 5.43) and systematic bias (*p*= 0.03) were demonstrated. For these teeth, the method tended to measure an average of 0.98° (0.10 - 1.86) less than the Invisalign^®^ Table.

When evaluating the inclination, results have shown high overall ICCs (> 0.90) and narrow limits of agreement (≤ 3°) for posterior teeth. Systematic errors were not found. Anterior teeth, however, presented limits of agreement with discrepancies higher than the clinically relevant difference. Angulation presented ICCs that were not always indicative of a great relationship, and the limits of agreement were not as interesting; additionally, systematic errors were present for premolars and molars.

Considering that the values for the random error (Dahlberg) were mainly less than 3°, and considering the clinical plausibility, only differences higher than 3° were considered relevant.

### RELIABILITY

The intra-examiner reliability is described in Tables 5 and 6, while the inter-examiner is described in Tables 7 and 8. The method was shown to be highly reliable for measuring the three kinds of movements, since the discrepancies were less than 1° for intra-rater, and less than 2° for inter-rater reliabilities. 

**Table 5:** ICC and Dahlberg index for ∆planned in the test-retest (intra-rater reliability).

	Rotation			Inclination			Angulation			
	ICC	95% CI	D	ICC	95% CI	D	ICC	95% CI	D	n
Maxillary and Mandibular teeth	1.00*	1.00-1.00	0.08	0.99*	0.99-1.00	0.16	0.98*	0.98-0.99	0.55	196

*p<0.05; ICC: Intraclass Correlation Coefficient; CI: Confidence Interval; D: Dahlberg.

**Table 6:** Descriptive statistics, 1-sample t test, and 95% CI values for differences and limits of agreement (positive numbers represent underestimation, and negative represent overestimation of measurements obtained by the test with respect to retest).

Differences (Retest - Test)	P value	Mean Difference (degrees)	SD (degrees)	95% CI (degrees)	Limits of agreement (degrees)	n
Rotation	0.47	-0.006	0.12	-0.02 - 0.01	-0.24 - 0.23	196
Inclination	0.32	-0.016	0.23	-0.05 - 0.01	-0.47- 0.43	196
Angulation	0.91	0.003	0.43	-0.06 - 0.06	-0.84 - 0.84	196

**Table 7:** ICC and Dahlberg index for ∆planned (inter-rater reliability).

	Rotation			Inclination			Angulation			n
	ICC	95% CI	D	ICC	95% CI	D	ICC	95% CI	D	
Maxillary and mandibular teeth	1.00*	0.99-1.00	0.15	0.96*	0.95-0.97	0.61	0.98*	0.98-0.99	0.74	196

*p<0.05; ICC: Intraclass Correlation Coeficient; CI: Confidence Interval; D: Dahlberg.

**Table 8:** Descriptive statistics, 1-sample *t* test, and 95% CI values for differences and limits of agreement (positive numbers represent overestimation, and negative represent underestimation of measurements obtained by the rater 2 with respect to rater 1).

Differences (Rater 2 - Rater 1)	P value	Mean difference (degrees)	SD (degrees)	95% CI (degrees)	Limits of agreement (degrees)	n
Rotation	0.54	0.009	0.22	-0.02 - 0.04	-0.42 - 0.44	196
Inclination	0.96	-0.002	0.68	-0.10 - 0.09	-1.33- 1.33	196
Angulation	0.75	-0.019	0.86	-0.14 - 0.10	-1.69 - 1.67	196

*p<0.05.

## DISCUSSION

In the present study, dental rotation, inclination and angulation were longitudinally obtained by calculating the ∆planned. To the best of our knowledge, this is the first study to test the validity and reliability of a method for longitudinal evaluation of teeth positioning using digital models.

Bearing in mind that correlation coefficients lower than 0.5, between 0.5 and 0.75, between 0.75 and 0.9, and higher than 0.9 are indicative of low, moderate, good and excellent relationships,^18^ respectively, it can be noticed that rotation measurements had excellent indexes for all groups of teeth (Tables 1 and 2). Similarly, the evaluation of inclination for premolars and molars also presented excellent values. The same results were not found for the inclination of anterior teeth and angulation of all teeth, which mostly presented low or moderate indexes.

The correct statistical approach to measure the degree of agreement between two methods is not obvious.^19^ ^,^ ^20^ It has been discussed that the ICC must not be considered alone for this analysis because high correlation does not necessarily mean that two methods agree.^19^ The Bland-Altman limits of agreement were applied to overcome this problem.

We presume that limits with discrepancies >3° are clinically relevant, and therefore consider the method as having limited value when longitudinally measuring the maxillary canine’s rotation, the inclination of anterior teeth and angulation of all teeth (Tables 3, 4). For the maxillary canines, for example, the method tends to underestimate the real rotation value up to 5.43°, or overestimate up to 3.47 (-3.47 - 5.43). In cases in which there is no problem in accepting this discrepancy, the method to perform this measurement can be applied.

The fact that the Table of Movements and the method yielded different results for some measurements might be related to limitations in the stability of the reference plane between the initial and final planned models. When Invisalign^®^ receives the initial digital models, or when they create it using impressions, the model has its own reference plane. It happens that probably a setup is performed on this model to generate the final model, and the ∆measurements are obtained in relation to this plane, which remains stable. The stability of the plane is guaranteed because the first file generates the second. Nevertheless, a problem is found when the task is needed in the opposite way: create a stable reference plane for the two files.

According to Ferrario et al.^21^, the stability of the plane is important for longitudinal analyses because the final measurements will be obtained in relation to a different reference. The sample in the present study was composed of individuals with a tendency towards an anterior open bite, and treatment planning included extrusion of anterior teeth, thereby rotating the plane in the anteroposterior direction. For this reason, instead of constructing two reference planes (one on the initial and other on the final model), we opted to make a “best fit” alignment of the teeth from both models and to construct a single plane as an attempt to attenuate this problem. Additionally, we opted to construct the plane differently from the recommendation of Richmond et al*.* ^15^, that have used the incisal edges and cuspid tips. Instead, we used the same approach as Huanca Ghislanzoni et al.^13^, who advocated that constructing the plane closer to the gingival region would make it less susceptible to changes caused by alterations in dental inclination, curve of Spee or Wilson. All these approaches, however, appeared to be insufficient for guaranteeing plane stability.

Although it cannot be affirmed, we believe that the reason for the great agreement of the inclination of posterior teeth, despite the instability of the plane, might be related to the process of obtaining this measurement. Because measurements were made in relation to the XZ plane, an alteration in the anteroposterior direction would compromise a measurement in the anterior region, but may not highly compromise measurements in the posterior region. However, an alteration in the lateral direction, as expected for patients who are treated for canted occlusal planes (not the case of the present sample), will probably affect the measurement in the posterior region and may not greatly compromise it in the anterior one. Regarding rotation, it must be remembered that while inclination and angulation were obtained in relation to the Z-axis, rotation was obtained in relation to the Y-axis. The positioning of this axis was not significantly changed by alteration in the reference plane, which was corroborated by the results of the present study, at least for small variances.

The use of a stable plane of reference, such as a cranial plane, could probably solve problems of instability. Digital models and tomographic images can therefore be superimposed, as performed by Castro et al.^22^ However, as mentioned before, CBCT is not indicated as a routine exam.^8^

For reliability, excellent ICCs and levels of agreement were achieved, with very low discrepancies for all kinds of movements. This fact is probably due to the consistency in transferring points from the initial to the final model. Nouri et al*.* ^14^ measured dental inclinations cross-sectionally in models by two means: manually, with the use of a tooth inclination protractor (TIP), and digitally, by means of *software* developed for this purpose. As a result, these authors verified that the digital method had a slightly higher intra-rater level of reliability than the manual method, with ICCs of 0.88, 0.76 and 0.87 for the incisors, canines and posterior teeth, respectively. Moreover, Kodaka et al.,^23^ who measured inclination manually, and Castro et al.,^7^ who measured inclination and angulation in digital manner, both cross-sectionally, obtained a total ICC of 0.98 for intra-rater reliability. Our results are similar to these results, since we achieved an ICC of 0.99 (0.99-1.00) for inclination, and of 0.98 (0.98-0.99) for angulation.

Although it is not the purpose of the study, one of the possible applications of this method is to quantify how predictable the digital planning of clear aligners is. The ∆planned and ∆achieved movements of patients undergoing treatment with aligners can therefore be compared. We believe that, in this specific situation, even the methods that presented limited validity could be applied. The explanation is that if both ∆s are calculated by the same method, any presumed error will most likely be maintained for both variations, making the comparison valid. Besides, the method proved to be highly reliable for all the measurements.

## CONCLUSIONS

The method developed is highly reliable for evaluating the three movements, and valid for the longitudinal evaluation of the rotation of all teeth (except maxillary canines) and the inclination of premolars and molars, even when a small anteroposterior change in the reference plane is present.

It has limited value to measure the rotation of maxillary canines, the inclination of incisors and canines or angulation of all teeth, at least in models in which an alteration in the overbite is present.
